# Aerosol Delivery of Polyelectrolyte Surfactant—Antimicrobial Nanoparticles to the Lungs

**DOI:** 10.1007/s11095-025-03985-2

**Published:** 2026-01-09

**Authors:** Yadiel Varela Soler, Amanda S. Padilla-López, Sughosha Rao, Leonardo Calderon, Gediminas Mainelis, Olga Garbuzenko, Tamara Minko, David I. Devore, Charles M. Roth

**Affiliations:** 1 Department of Chemical and Biochemical Engineering, Rutgers University, Piscataway, NJ 08854 USA; 2 Department of Biomedical Engineering, Rutgers University, Piscataway, NJ 08854 USA; 3 University of Puerto Rico, Mayaguez, PR 00680 USA; 4 Department of Environmental Sciences, Rutgers University, New Brunswick, NJ 08901 USA; 5 Department of Pharmaceutics, Rutgers University, Piscataway, NJ 08854 USA; 6 Graplon Technologies, LLC, Langhorne, PA 19047 USA

**Keywords:** antimicrobial, biodistribution, nanoparticle, nebulization, pulmonary delivery

## Abstract

**Background:**

Lung infections affect over 80% of adults with cystic fibrosis, with *Pseudomonas aeruginosa* being a leading pathogen. Although antibiotics are frequently nebulized as standard treatments, the physicochemical environment of the diseased lung often limits their diffusion and overall effectiveness. Our previous studies showed polyelectrolyte surfactants (PS) to be a promising delivery system for cationic antimicrobials *in vitro*. This study seeks to expand that knowledge by evaluating their potential for nebulized delivery.

**Methods:**

To achieve this, we evaluated their size and antimicrobial activity following nebulization; *in vitro* toxicity against epithelial cells and erythrocytes; and biodistribution and expression of inflammation markers following administration to healthy mice.

**Results:**

The nanoparticle formulation exhibited a mucolytic effect on an artificial mucus model of cystic fibrosis mucus. Following nebulization, nanoparticles retained both their size and biological activity. Additionally, they displayed no observable toxicity *in vitro* against either human lung epithelial cells or erythrocytes; instead, epithelial cells treated with PS-based nanoparticles showed increased cell viability. Following administration of these formulations to mice via inhalation, over 70% of the recovered nanoparticles were retained in the lungs 24 h after treatment, with a small fraction being uniformly distributed to other tissues. A screen of key inflammatory cytokines revealed that inhalation treatment led to a slight increase of IL-6 in the liver and IL-18 in the spleen. These increases seem to be consistent with a minor inflammatory response.

**Conclusion:**

Overall, the results suggest that PS are a promising nanotechnology for the pulmonary delivery of cationic drugs.

**Supplementary Information:**

The online version contains supplementary material available at 10.1007/s11095-025-03985-2.

## Introduction

In 2021, the Behavioral Risk Factor Surveillance System (BRFSS) survey reported that approximately 33.8 million adults in the U.S. were affected by chronic lung disease, such as lung cancer, cystic fibrosis (CF), and asthma [1]. All of these conditions have pharmaceutical treatments available, whose therapeutic efficacy depends on achieving high drug concentrations in the lung. However, less than 25% of the treatment dose is typically retained in the lung following systemic delivery [2]. As a result, inhalation is the preferred delivery method for therapeutics targeting chronic lung disease. Inhalation has been employed for delivery of bronchodilators, mucus thinners and antimicrobials using devices that include nebulizers, metered-dose inhalers, and dry powder inhalers [3, 4]. The lungs possess a large surface area for deposition and high vascularization, making them an attractive route of administration [5]. Additionally, by directly targeting the lungs, drugs can bypass degradation in the gastrointestinal tract and the first-pass effect in the liver.

In the case of CF, a primary cause of morbidity is infection with bacterial pathogens, including *Pseudomonas aeruginosa*, for which ~ 50% of adult patients test positive [6]. Although antimicrobial agents such as tobramycin reduce the disease burden associated with chronic *P. aeruginosa,* they often fail to fully eradicate the infection. As a result, over 80% of patients suffer from persistent bacterial lung infections [7]. Furthermore, infections that are not successfully cleared have a greater likelihood of developing into drug-resistant strains. The reduced efficacy is due to challenges in drug distribution within the lungs, combined with the biophysical barriers imposed by excessive CF mucus and bacterial biofilms. Given the limitations of current treatments, there is great interest in developing novel antimicrobials and enhancing the efficiency of existing therapies via advanced formulations.

Inhaled tobramycin is typically the preferred treatment against chronic *P. aeruginosa* infections, with inhaled polymyxins serving as last-resort medications. However, the effectiveness of these drugs is often limited by poor drug transport and rapid degradation within the lung environment. One method to overcome these limitations is through the use of nanotechnologies, which have shown the capacity to control release [8–11], evade the host-immune response [12], and limit drug degradation [9, 13, 14]. The recent FDA approval of Arikayce®, a liposomal amikacin formulation for pulmonary delivery, highlights the potential of nanoparticle-based drug delivery systems to improve the efficacy of inhaled therapies. Arikayce® is currently approved as a last-line therapy for *Mycobacterium avium complex* infections [15]. Other inhaled antimicrobial nanotechnologies have been developed, such as Apulmiq®, a liposomal ciprofloxacin suspension for *P. aeruginosa* infections in non-CF bronchiectasis. However, clinical trial results have not yet been definitive [16].

We have previously developed antimicrobial-loaded polyelectrolyte surfactant nanoparticles for the treatment of lung infections, which showed promising activity against *P. aeruginosa* *in vitro* [17]. In these studies, tobramycin and polymyxin B were each incorporated into self-assembled nanoparticles formed by combining drug with a polyelectrolyte surfactant. Our previous work identified lead polyelectrolyte surfactants that are composed of an anionic poly(methacrylic acid) (PMAA) backbone, grafted with various percentages of polyetheramine (Jeffamine M-2070, J) pendant chains. We refer to these as PMAA-g-X%J, where X stands for the target graft percentage.

To apply these nanoformulations for pulmonary delivery, it is important to characterize their impact on mucus viscosity, as well as to evaluate the effect of nebulization on their physical properties and biological activity, and on their physiological disposition after undergoing nebulization. In this study, we evaluated particle size and antimicrobial activity before and after nebulization, cytotoxicity in human erythrocytes and bronchial epithelial cells, and biodistribution profiles in mice following pulmonary and systemic delivery. To further assess the *in vivo* response, we also measured levels of inflammatory cytokines—including IL-6, IL-18, and TNF-α—across relevant tissues.

## Materials and Methods

### Nanoparticle Preparation

The polyelectrolyte surfactants (PS) were synthesized as previously described [17]. Briefly, for PMAA-g-10%J, PMAA (Polyscience Inc., Warrington, PA), 2.3 mmol of repeat units, 0.23 mmol of hydroxybenzotriazole (HOBt; MilliporeSigma, Burlington, MA) and 0.24 mmol of Jeffamine M-2070 (Huntsman Corp., Woodlands, TX) were dissolved in 3 mL of Dimethylformamide (DMF; MilliporeSigma, Burlington, MA). Then 0.23 mmol of N,N-Diisopropylcarbodiimide (DIC; MilliporeSigma, Burlington, MA) is added and allowed to react for 30 h at 50 °C. DMF was removed by evaporation and the PS resuspended in methanol. Dissolved polymer was precipitated by adding dropwise to diethyl ether (20 mL; MilliporeSigma, Burlington, MA) and then recovered via filtration and allowed them to dry under vacuum. For PMAA-g-5%J, the process was the same, but half the amount of Jeffamine M-2070 was used. Nebulization and biodistribution studies were performed with PMAA-g-10%J tagged with 0.65% of the fluorescent dye Cy5.5, as previously described [17], to allow for detection.

As detailed in our prior publication [17], nanoparticles (NPs) were prepared by combining PS and tobramycin or polymyxin B (MilliporeSigma, Burlington, MA) at a charge ratio of approximately 0.5. This ratio was chosen based on prior studies that showed good colloidal stability and high encapsulation efficiency [17, 18]. For NP formulation, polymers were sonicated for 20 min on ice and then stirred on ice on a magnetic stir plate. The appropriate amount of antimicrobial was added dropwise, and the NPs were left to mix for 10 min. Lastly, the NPs were sonicated for 20 min on ice. NPs were prepared fresh on the day of each experiment.

### Artificial Mucus Preparation

An artificial mucus model was prepared to mimic CF mucus, for which the formulation was adapted from prior studies [19–21]. The initial model consisted of calf thymus DNA (5 mg/mL), porcine gastric mucin type II (30 mg/mL), magnesium chloride (1.2 mM), potassium chloride (150 mM), calcium chloride (0.2 mM), tris base (22 mM), glucose (3.2 mM), and mixed casamino acids (3 mg/mL) dissolved in phosphate buffered saline. All reagents were purchased from MilliporeSigma (Burlington, MA). The mixture was adjusted to pH 7 before the addition of DNA and, once dissolved, adjusted again to pH 6.8. Undissolved portions of DNA were homogenized into the mixture by passing through a needle and syringe.

### Rheology

To assess the effect of our polymers on mucus viscosity, rheology studies were performed. Artificial mucus (1 mL) was combined with compounds of interest (100 µL), and a shear-up ramp (10–600 s^−1^) was carried out at 37 °C using an Anton Paar MCR 302e Rheometer (Graz, AT) in a cone-plate geometry. Nanoformulations or polymer alone were evaluated at a concentration of 8 mg/mL, which approaches the solubility limit for our polymer. Other compounds evaluated include the nonionic surfactant Triton X-100 (TX 100, 10% v/v) and mucolytics N-acetylcysteine (NAC, 10%w/v) and DNase I (200 U/mL) [22, 23]. All treatment groups were incubated with mucus at room temperature for at least 15 min before rheology testing.

### Nebulization of Nanoparticles

Initial testing of nanoparticle stability following nebulization was conducted using the clinically approved PARI PRONEB® MAX aerosol delivery system (Midlothian, VA). Nanoparticles were evaluated for their size, mass recovery, and minimum inhibitory concentration (MIC) against *P. aeruginosa* before and after nebulization. Briefly, the nebulizer mouthpiece was connected to a 200 mm Graham condenser cooled with ice water. Two milliliters of formulation were loaded into the nebulizer and nebulized until the chamber was fully emptied. The resulting aerosol was collected by condensation. The size was determined using dynamic light scattering (DLS; Wyatt DynaPro® Plate Reader III, Santa Barbara, CA), and the mass of polymer via fluorescence measurements using a microplate reader. The mass of drug was determined using a high performance liquid chromatography (HPLC) system (Agilent 1260 Infinity II, Santa Clara, CA) as previously described [17].

### Minimum Inhibitory Concentration (MIC)

The commercially available *Pseudomonas aeruginosa* strain PAO1 (ATCC15692) was grown in trypticase soy broth (TSB) (BD, Franklin Lakes, NJ). Bacterial density in overnight cultures was determined by measuring the optical density at 600 nm and diluting to 10^7^ CFU/mL. Each well in a 96-well plate was inoculated with 10^6^ CFU, followed by the addition of 100 μL of treatment. Treatment conditions included serial dilutions of nebulized and non-nebulized NPs. Well plates were incubated overnight at 37 °C. The MIC was defined as the lowest concentration capable of inhibiting visible bacterial growth.

### Aerosol Droplet Size Characterization

Aerosol droplet size was evaluated following nebulization of PMAA-g-10%J:PB nanoparticles with a 3-jet Collison nebulizer (CH Technologies, Westwood, NJ) at an airflow rate of 5 L/min and operating pressure of 20 psig. A HEPA-filtered airflow of 80 L/min diluted and desiccated the aerosol droplets. The size distribution of resulting particles was measured with a system comprising a Scanning Mobility Particle Sizer 3986 (SMPS) and an Aerodynamic Particle Sizer 3321 (APS), which allowed us to span both nano- and micro- size regimes (14–20,000 nm) [24, 25]. The size distribution data were combined using Merge software (TSI, Inc.). Polyvinyl alcohol (PVA) and hydroxypropylmethylcellulose (HPMC) were added to improve suspension stability and reduce surface tension. The background air circulated inside a bio-hood with HEPA filters, and Mili Q water was used as a reference.

### CFBE41o-Cell Culture

CF human bronchial epithelial (CFBE41o-) cells were grown in minimum essential medium eagle (MEM) supplemented with 10% fetal bovine serum (FBS), 2 mM L-glutamine, and 1% penicillin/streptomycin at 37 °C and 5% CO_2_. The cells and supplies were purchased from MilliporeSigma (Burlington, MA). Once cells reached approximately 90% confluency, they were detached by trypsinization (ATCC, Manassas, VA) subcultured, used for cell viability assays, or cryostocked with DMSO as a cryoprotectant.

### Metabolic Activity Assay

The cytotoxicity of NP formulations on CFBE41o- cells was determined via a 3-(4,5-dimethylthiazol-2-yl)−5-(3-carboxymethoxyphenyl)−2-(4-sulfophenyl)−2H-tetrazolium, inner salt (MTS) assay (Promega, Madison, WI). Cells were seeded in a 96-well plate at a density of 10,000 cells per well (100 μL) and incubated for 24 h. The cells were then treated with 100 μL of each formulation and incubated for an additional 24 h. Once at the end, 20 μL of the MTS assay solution was added to the plate, it was incubated for 1 h at 37 °C, and the absorbance was read at 490 nm using a microplate reader (Spectramax M3, Molecular Devices, Sunnyvale, CA). Cell metabolic activity was expressed relative to the PBS treatment control. Cells were treated at a drug concentration of 300 μg/mL, be it drug alone or encapsulated. Likewise, the corresponding polymer concentration of 4.65 mg/mL was used for both polymer-only and nanoparticle treatments.

### Hemolysis Assay

Human red blood cells (RBC) from were kindly gifted by Prof. Valerie Tutwiler (Department of Biomedical Engineering, Rutgers University, NJ). 2 mL of PBS (pH 7) were added to the vial containing RBC and gently inverted until fully resuspended. The vial was centrifuged at 1,700 rcf for 5 min and the supernatant removed. Washing of RBC was repeated four times, in order to remove free hemoglobin. After washing a 1% RBC suspension was prepared in PBS. In a 96-well plate, 100 μL of treatment or controls were added to each well, followed by 100 μL of the 1% RBC suspension. Samples were then incubated for 1 h at 37 °C. Plates were centrifuged at 1,700 rcf for 5 min, and 100 μL of the supernatant were transferred to a new 96-well plate. Finally, the absorbance was measured at 405 nm in a microplate reader. PBS and 1% Triton X-100 (Promega, Fitchburg, Wisconsin) were used as negative and positive controls, respectively. Hemolysis ratio (HR) was obtained from normalizing the sample absorbance to that of the negative and positive controls [26]. Treatment concentrations were the same ones used for viability studies.

### Biodistribution Study

The animal use protocol was approved by the Rutgers Institutional Animal Care and Use Committee under ID# TR201800056. A cohort of 5–7 week old SKH-1 hairless female mice was treated with Cy5.5-tagged PMAA-g-10%J:TB nanoparticles in order to evaluate the *in vivo* biodistribution of nanoparticles and signs of acute toxicity. Mice were administered treatment directly to the lungs via inhalation or systemically via intraperitoneal (IP) injection. The treatment groups for each delivery route included: PBS, drug alone, polymer alone, and NPs. Groups receiving PBS as their treatment consisted of four mice, while the other treatment groups consisted of six mice each. Inhalation delivery was performed following previously described procedures [25]. Briefly, a one-jet Collison nebulizer (CH Technologies) was used to aerosolize at a flow rate of 2 L/min. The aerosol was then delivered to each animal containment tube for 10 min; the animals’ noses were placed directly on the spout delivering the aerosol. Openings at the back of the tubes allowed exhaled air to escape. Nanoparticle formulations for both the inhalation and IP groups contained a TB concentration of 90 μg/mL and a PMAA-g-10%J concentration of 1.4 mg/mL, and each mouse was treated with a volume of 200 μL. Twenty-four hours after treatment, mice were sacrificed and tissues were imaged using an IVIS (Xenogen, Alameda, CA, USA) system. The images were then analyzed for fluorescence intensity using ImageJ. After imaging, tissues were stored at −80 °C until further use. Whole tissues were homogenized in cell extraction buffer (Invitrogen, Thermo Fisher Scientific, Waltham, MA, USA) supplemented with 1.5% protease inhibitor cocktail (MilliporeSigma, Burlington, Massachusetts) using 1 mL of buffer per 100 mg of tissue. The lysate was then centrifuged at 13, 000 × *g* for 10 min at 4 °C and the supernatant transferred to a new tube.

### Multiplex Assay

An initial assessment of inflammatory cytokines was performed on a subset of tissues using a ProcartaPlex™ cytokine panel (Cat: EPX110-20820–901, Thermo Fisher Scientific, Waltham, MA). Total protein concentration in each tissue lysate was quantified using a Pierce BCA assay kit (Thermo Fisher Scientific, Waltham, MA) according to manufacturer specifications. The lysate from each tissue was then diluted to an equal concentration for multiplex assays. Multiplex assays were performed at the Rutgers Cancer Institute, Immune Monitoring and Flow Cytometry (IMFC) Shared Resource Core Facility. The inflammatory cytokines detected by the panel were GM-CSF, IFN-γ, IL-1β, IL-2, IL-4, IL-5, IL-6, IL-12, IL-13, IL-18, and TNF-α.

### ELISA assays

Following the multiplex screening, enzyme-linked immunosorbent assay (ELISA) was used to measure specific cytokines of interest in all collected tissues and mouse replicates. IL-6 and TNF-α concentrations in tissue homogenates were determined using ELISA kits from Biolegends (Cat. No. 431304 and 430,904, respectively) (San Diego, CA). IL-18 concentration was measured using ELISA kits from R&D Systems, Cat. No. DY7625-05 (Minneapolis, MN).

### Statistical Analysis

Data were analyzed using standard unpaired Student t-test for two groups, or one-way and two-way analysis of variance (ANOVA) for multiple groups. The Tukey’s and Šídák’s pairwise comparison tests were used for one-way and two-way ANOVAs, respectively. Assumption of normal distribution and equal variances were used. All measured quantities are represented by their mean value, and their error bars by their standard error of the mean. Statistical significance was evaluated using an alpha value of 0.05. Plots show statistical significance using the following notation: * for p < 0.05, ** for p < 0.01, *** for p < 0.001, and **** for p < 0.0001. All statistical testing and plotting were performed in GraphPad Prism.

## Results and Discussion

### Mucus Thinning Effect

**Fig. 1:**
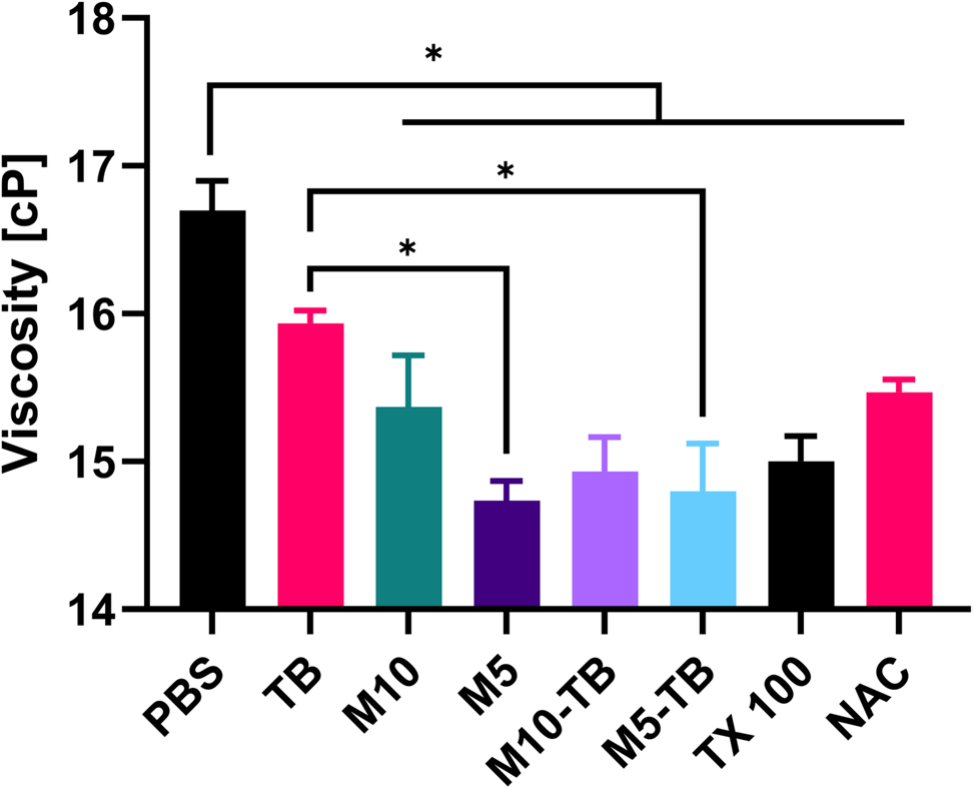
Effect of formulations on artificial mucus viscosity at 500 s^−1^. Each test consisted of 1 mL of mucus and 0.1 mL of treatment. Nanoformulations and polymers alone were tested at 8 mg/mL, while the surfactant Triton X-100 (TX 100) and mucolytic N-acetylcysteine (NAC) were evaluated at 10%v/v and 10%w/v, respectively. The polymers PMAA-g-5%J and PMAA-g-10%J are denoted as M5 and M10, respectively. Bars represent the average and standard error of the mean. *p < 0.05.

An important barrier to effective pulmonary drug delivery is the non-uniform distribution of drugs due to the viscous nature of airway mucus. In cystic fibrosis (CF), patients are often prescribed nebulized mucolytics to reduce mucus viscosity, promoting airway clearance and improving drug transport. Using an artificial CF mucus model, we evaluated the mucolytic potential of our nanomedicine. Mucus showed a shear thinning behavior, consistent with previous observations in the literature (Figure S1) [27]. The two mucus components that contribute the most to its rheological behavior are mucins and DNA [19]. Addition of N-acetylcysteine (NAC; 10%w/v), a commonly used mucolytic agent, produced a significant decrease in viscosity relative to the PBS control (Fig. 1). This effect, while statistically different, was less than that observed for most other tested formulations. The low effect of NAC on mucus viscosity suggests that mucins, NAC’s primary target via disulfide bond reduction [28], play a relatively minor role in the viscosity of the mucus. Additionally, while the concentration of NAC used is comparable to that of clinical inhalation solutions, studies have suggested that higher doses may be necessary for a noticeable reductive effect [28]. While treatment with NAC did not exert a major effect on rheological behavior, treatment with DNase I (200 U/mL) significantly reduced viscosity of the mucus (Figure S1), supporting the premise that the DNA makes a significant contribution to mucus rheology. Dornase alpha (Pulmozyme®), a recombinant human DNase I, is typically prescribed to CF patients as it allows for DNA degradation and thus reduction in mucus viscosity, which in turn facilitates airway clearance [22]. These results suggest viscosity was primarily attributed to DNA concentration and molecular weight; in support of this, mucus prepared with the lower molecular weight, salmon sperm DNA, exhibited minimal viscosity (data not shown).

Addition of free drug resulted in a minimal, not statistically significant, drop in viscosity (Fig. 1). On the other hand, polyelectrolyte surfactants (PS; 8 mg/mL), both alone and in combination with tobramycin (TB), induced a statistically significant decline of roughly 10% in mucus viscosity, relative to the PBS control. The effect of our PS was comparable to that of the stronger, commercially available nonionic surfactant Triton X-100 (10%v/v). Surfactants are thought to decrease viscosity by increasing the solvation of hydrophobic components within the mucus [29]. These approximately 10% reduction in mucus viscosity by nanoformulations, combined with other features like controlled release and decreased drug degradation, could promote improved drug delivery across the mucus matrix barrier.

### Evaluating NP Biophysical Stability After Nebulization

**Fig. 2:**
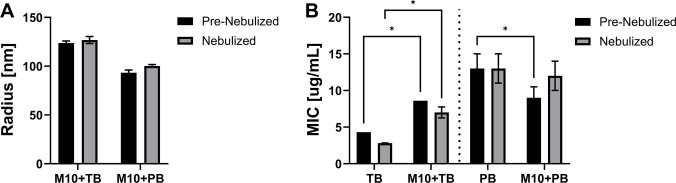
Tobramycin (TB) and polymyxin B (PB) loaded nanoparticles were evaluated for their (**A**) size and (**B**) antimicrobial activity pre- and post-nebulization. Nanoparticles were prepared with the graft copolymer PMAA-g-10%J, here designated as M10. No statistically significant difference in size was observed among the tested conditions. Bars represent average and standard error of the mean, *n* = 4. *P < 0.05.

Pulmonary delivery of nanoparticle formulations is often limited by their ability to aerosolize without significantly affecting nanoparticle properties. We used the PARI PRONEB® MAX nebulizer, used clinically by CF patients, to aerosolize our nanoparticles, after which we evaluated their physical stability. The nanoparticles were formed from PMAA-g-10%J (molecular weight 290 kDa) polyelectrolyte surfactants, encapsulating one of the cationic drugs, polymyxin B (PB; 160 µg/mL) or tobramycin (TB; 69 µg/mL). Following nebulization, aerosolized nanoparticles were collected by condensation. Nebulization induced no detectable change in size distribution, with collected nanoparticles retaining a size average of approximately 200 nm diameter (Fig. 2A). Minimum inhibitory concentration (MIC) assays performed with the collected samples showed that the nanoformulation retained the same level of antimicrobial activity before and after nebulization (Fig. 2B). We did note that TB-loaded NPs experienced a small increase in MIC against PAO1 culture compared to free TB alone. In contrast, PB nanoformulations showed an increase in antimicrobial activity, consistent with previous findings for these formulations [17]. It should be noted that, although the MIC values were highly reproducible within our studies, they were higher than those typically reported in literature for PAO1 [30, 31]. It has been noted by others that MIC values are sensitive to assay conditions, particularly media composition. The TSB media used in this work tends to produce higher MIC values [32], as observed here.

A roughly 50% loss of both drug and polymer mass in the collected suspension was observed (**Table S1**), as determined with HPLC and UV detection. The mass loss likely occurs across multiple sources, including the nebulizer mesh, inner condenser walls, and possibly entrained in escaped vapor. The loss of nanoparticle components within nebulizers has been previously observed in literature [33]. However, it did not result in a drastic change of the physical properties or biological activity of these nanoparticles.

### Size of Aerosol Droplets

**Fig. 3:**
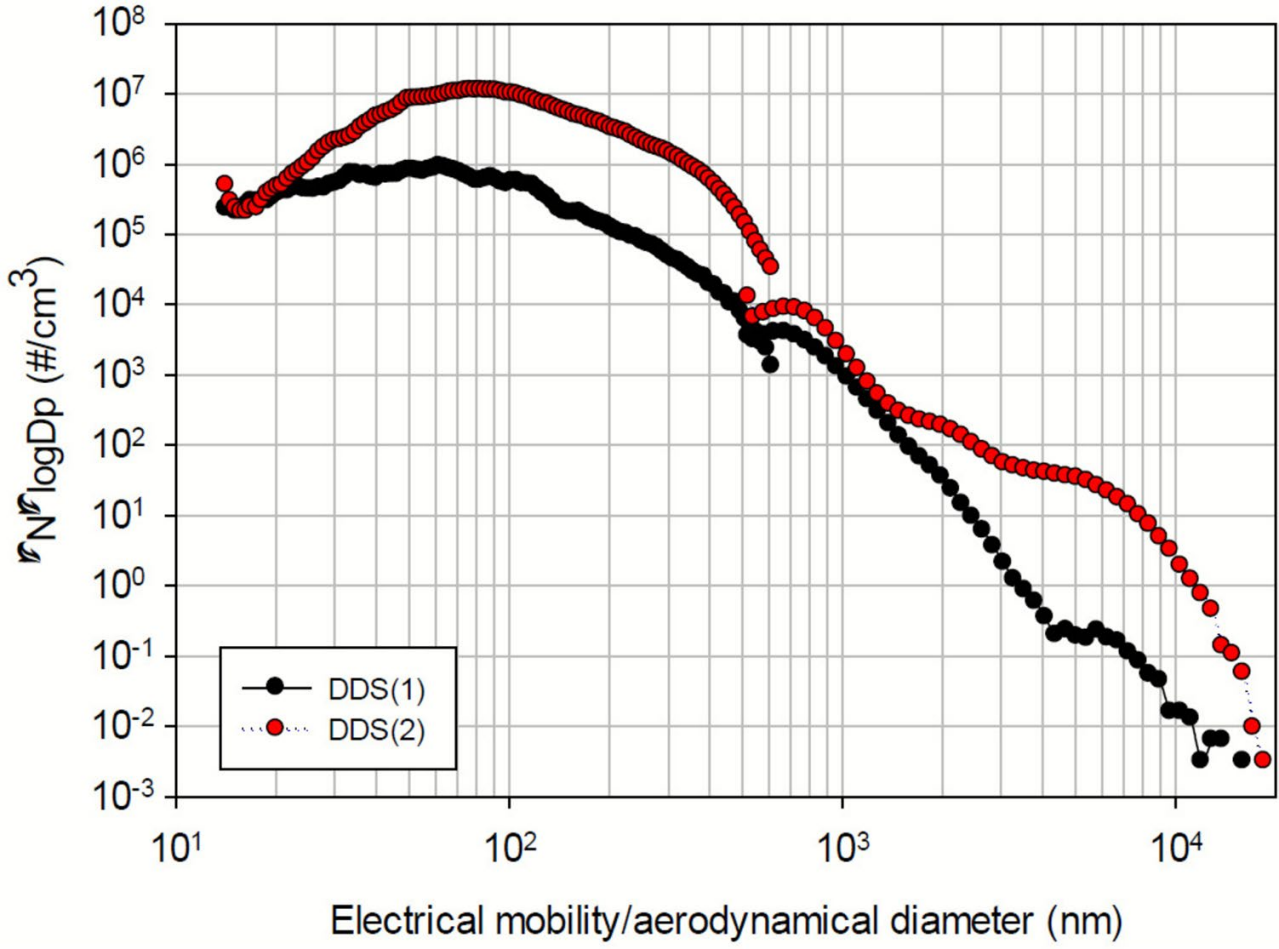
Log-normal size distribution of aerosolized PMAA-g-10%J:PB nanoparticles using a 3-jet Collison nebulizer. Electrical mobility diameters between 10–700 nm were determined via SMPS and aerodynamic diameters between 0.5–10 μm were determined via APS. (In the overlapping region, data from both instruments are shown). Shown in black, named DDS(1), refer to aerosolized droplets that passed through a desiccation chamber. In red (DDS(2)) display droplet size with no desiccation.

Aerosol droplet size is an important feature as it plays a major role in pulmonary deposition of inhaled therapies, with droplets above 5 μm in diameter remaining trapped in the upper airways. Here we evaluated the droplet size distribution produced from the nebulization of PMAA-g-10%J:PB nanoparticles with a 3-jet Collison nebulizer (Fig. 3). Results show a polydisperse size distribution with particle sizes ranging from 14 nm to 20,000 nm, reflecting the presence of nanoparticles and various other components, like salts, in the nebulizer liquid. The vast majority of particles were less than 5 μm in diameter, making them suitable for deposition deep into the lungs.

### *In Vitro* Cytotoxicity of NPs

**Fig. 4:**
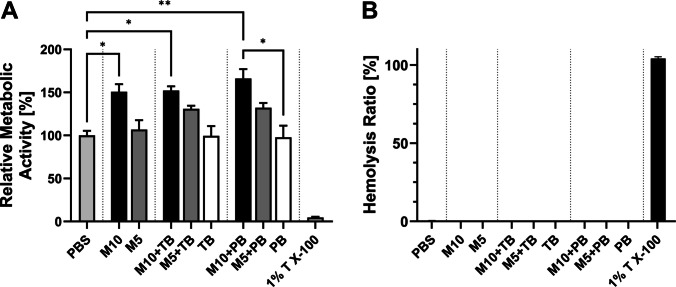
Cytotoxicity of the different formulations. (**A**) Cystic fibrosis human bronchial epithelial cells (CFBE41o-) were treated with different formulations of drug, polymer or in combination for 24 h, after which metabolic activity was determined using an MTS assay and normalized to the PBS control. (**B**) Hemolytic toxicity was evaluated by combining human red blood cells with the same formulations. Following a 1 h incubation, the amount of released hemoglobin was measured. The hemolysis ratio represents the ratio of released hemoglobin relative to the controls PBS and 1% Triton X-100. Bars represent the average and standard error of the mean, *n* = 3. *p < 0.05 and **p < 0.01.

Because bronchial epithelial cells cover the surface of our lungs, they are exposed to the highest concentration of drugs following pulmonary delivery. To assess the cytotoxicity of our previously developed nanoparticles on a relevant *in vitro* model, cystic fibrosis bronchial epithelial cells (CFBE41o-) were treated with formulations (300 μg/mL TB; 4.65 mg/mL PMAA-g-10%J or 2.94 mg/mL PMAA-g-5%J) and incubated for 24 h. After incubation, cell metabolic activity was measured using an MTS assay. Compared to the PBS-treated control group, cells treated with formulations containing PMAA-g-10%J saw a significant increase in metabolic activity (Fig. 4A). In contrast, formulations containing PS at a lower graft density (PMAA-g-5%J) saw a modest, but not statistically significant, increase in metabolic activity. At the concentrations tested, treatment with the drug alone showed no impact on cell metabolic activity. These observations align with prior studies indicating that grafted polymers can enhance cell proliferation [34–37]. Polymers such as Pluronic F-68, an amphiphilic copolymer made up of poly(ethylene oxide) and poly(propylene oxide), like Jeffamine, similarly were shown to have a positive effect on growth [38]. Furthermore, this effect seems to be dependent on graft density, as PMAA-g-10%J exhibited a more pronounced effect relative to its lower grafted counterpart. We speculate that the amphiphilic Jeffamine chains may interact with the cell membrane, stabilizing it and enhancing cell growth [39].

Once deposited in the lung, drugs are absorbed, at least to some extent, into the bloodstream due to the highly vascularized nature of the lungs. To evaluate potential hemolytic toxicity, we performed a hemolysis assay using human red blood cells exposed to nanoparticles at the same concentrations tested in the MTS assay. Hemolysis was quantified by measuring the absorbance of released hemoglobin relative to the positive and negative controls. No significant hemolysis was observed in any of the treatment groups (Fig. 4B), demonstrating that the PS nanoparticles exhibit no toxicity against erythrocytes.

### Biodistribution of NPs *In Vivo*

**Fig. 5:**
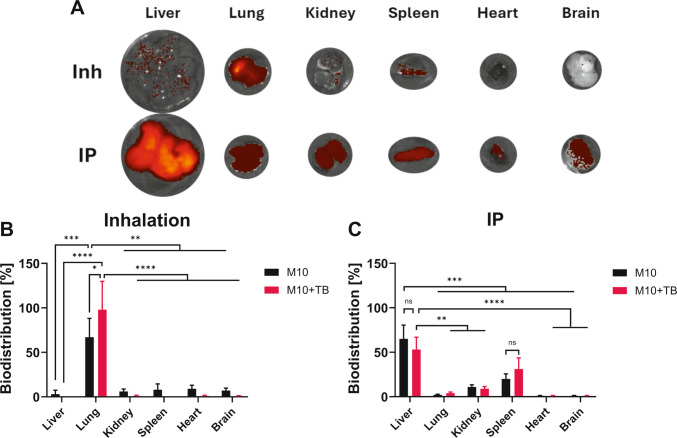
Mice were treated with Cy5.5 tagged PMAA-g-10%J-TB NPs via inhalation or IP injection. Tissues were harvested 24 h after treatment and imaged with an IVIS system (**A**, representative images). The mean fluorescence intensity of images from mice receiving treatment by (**B**) inhalation and (**C**) IP injection was analyzed with ImageJ and used to determine the relative biodistribution. Biodistribution was defined as the amount of fluorescence in the tissue of interest relative to that in all tissues combined. Background fluorescence was corrected using tissues from PBS treated animals. Bars represent average and standard error of the mean, *n* = 6. *p < 0.05, **p < 0.01, ***p < 0.001, and ****p < 0.0001.

Pulmonary delivery of antimicrobials is often preferred for patients with compromised lung function, as it provides direct access to the target site and can be administered along with other nebulized therapeutics to treat their lung symptoms. To evaluate the biodistribution and *in vivo* toxicity profiles, we treated mice with Cy5.5 tagged PMAA-g-10%J-TB nanoparticles (90 μg/mL TB; 1.4 mg/mL PMAA-g-10%J) delivered systemically via intraperitoneal (IP) injection or via inhalation with a jet nebulizer [25]. The drug concentrations used here are less than would be used for treatment of lung infections; they were selected to provide a robust but non-saturating imaging signal. Twenty-four hours after treatment, tissues were harvested from key organs and imaged to assess biodistribution of nanoparticle formulations following either administration route (Fig. 5). NP delivery by IP injection resulted in nearly 60% of the distributed dose being trapped in the liver, with another ~ 25% and ~ 10% in the spleen and kidneys, respectively, while only 1% of the distributed dose reached the lungs (Table S2). Similar distribution profiles have been observed for injected nanoparticles of a wide variety of chemistries [40–43], highlighting the role of the reticuloendothelial system in clearance of nanoparticles. This results in significant off-target accumulation following systemic delivery.

On the other hand, for mice that were treated via inhalation, over 70% of the recovered NP dose was retained in the lungs, while the remainder was distributed across the other organs (Table S2). For the nanoformulation containing tobramycin, 96% of the fluorescence was associated with lungs, with no more than 2% in any other organ. The increased retention in the lungs demonstrates the benefit of pulmonary delivery for nanoparticles, as it allows focused release of the drug directly to the lung while limiting systemic exposure and associated side effects. Most tissues showed no statistically significant difference between NPs and polymer alone, with the exception of the lungs following inhalation. We have previously observed differences between NPs and polymer alone in interactions with cells, and thus it is not surprising that they would exhibit differential retention in the lungs. A possible mechanism is that nanoparticle formulations have a lower net negative charge than polymer alone, resulting in less repulsion from anionic components of the mucus and epithelium, leading to being retained in the lung for longer. Due to limitations of the inhalation setup, a large portion of the nebulized dose escapes the inhalation chamber; as a result, mice only receive a small portion of the total dose. Studies with a similar setup suggest that the inhaled fraction could be as low as 0.5% of the intended delivery dose [25]. In contrast, mice receiving IP injections get the full 200 μL dose, necessitating that comparison between the two administration routes be normalized to the amount of fluorescently labeled polymer detected, rather than by dose. These observations demonstrate that nebulization of our polyelectrolyte surfactant nanoparticles leads to lung retention over the course of 24 h, with minimal leakage into the systemic circulation.

### Tissue-Specific Cytokine Expression

**Fig. 6:**
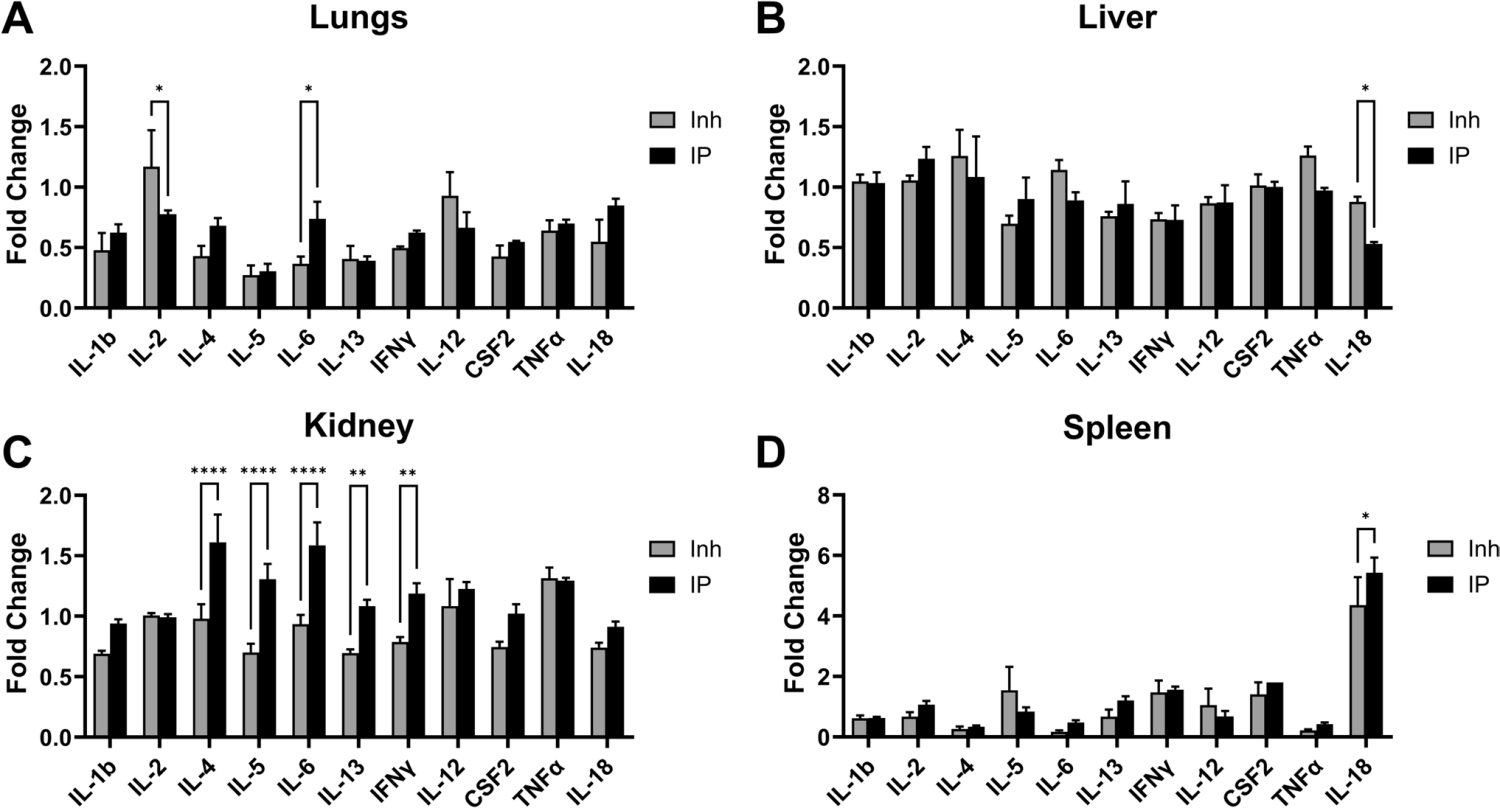
Expression of various cytokines in the (**A**) lungs, (**B**) liver, (**C**) kidney, and (**D**) spleen relative to no treatment mice 24 h after treatment with Cy5.5 tagged PMAA-g-10%J-TB nanoparticles by inhalation or IP injection. Bars represent the average and the standard error of the mean, *n* = 4. *p < 0.05, **p < 0.01, ****p < 0.0001.

After treatment, no mice showed obvious signs of discomfort or acute toxicity. To evaluate the tolerability of treatment more broadly, an initial screening was performed to measure the expression of 11 well-recognized inflammatory cytokines (Fig. 6). Cytokine levels in neither the lungs nor the liver were elevated compared to untreated mice following either treatment. In fact, a slight decrease in expression for most of the cytokines was observed. In the kidneys, IL-4, IL-5, and IL-6 were slightly upregulated only in mice treated by IP injection. TNF-α was upregulated in the kidneys following both IP and inhalation delivery. However, the extent of elevation for each of the four cytokines was 50% or less, which suggests that minimal inflammation is occurring when compared to the > twofold increases caused by other known drugs [44, 45]. In the spleen, a significant increase in IL-18 expression was observed for both delivery methods. This overexpression could be due to the recruitment of natural killer (NK) cells in response to the presence of NPs. While no significant levels of inflammation were noted in other tissues, the increased recruitment of NK cells due to nanocarrier exposure has been previously observed [46]. Additionally, increased levels of IL-18 signaling have been associated with a reduced number of NK cells in the spleen [47, 48].

**Fig. 7:**
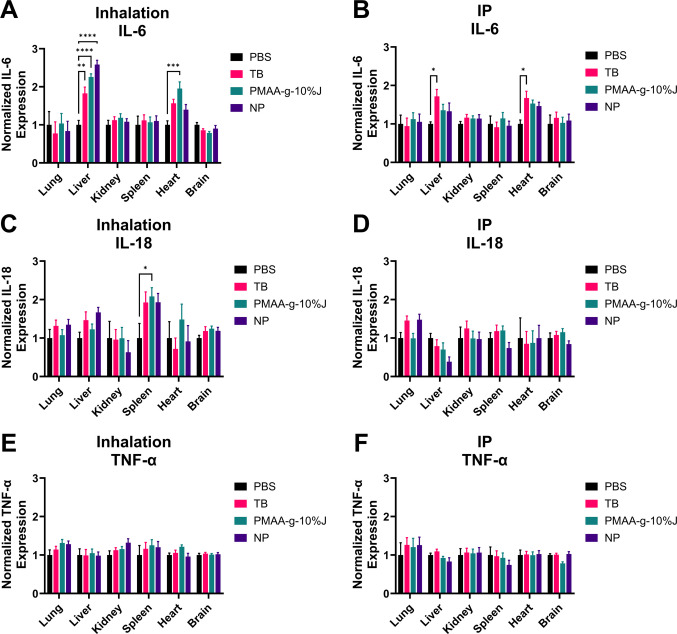
ELISA were used to determine the concentration of various cytokines in various tissues following pulmonary or systemic administration. IL-6 levels following (**A**) inhalation and (**B**) IP show a slight increase in both the liver and heart. Expression of IL-18 only showed increased levels in the spleen of mice dosed by (**C**) inhalation and not by (**D**) IP. TNF-α, on the other hand, didn’t show any increased expression regardless of the route of administration, (**E**) inhalation or (**F**) IP. Bars represent the average and the standard error of the mean, *n* = 6. *p < 0.05, **p < 0.01, ***p < 0.001, and ****p < 0.0001.

The increased expression of IL-6, IL-18, and TNF-α observed in our initial multiplex screen prompted us to further evaluate the expression of these inflammatory markers in all collected tissues and treatment groups. Therefore, we used more sensitive enzyme-linked immunosorbent assays (ELISAs) to determine their respective concentrations. IL-6 was significantly overexpressed in the liver following inhalation for all treatment groups, whereas it was increased following IP administration only for tobramycin alone (Fig. 7A-B). Lung and liver pathology have been shown to be strongly connected [49–51]. It is possible that inhalation of nanoparticles triggered a local immune response which subsequently circulated to the liver and induced a hepatic immune reaction. Importantly, the observed 2- to 2.5-fold increase is still below those normally associated with hepatotoxicity from known liver damaging drugs [44, 47, 52–55]. Increased levels of IL-6 in other tissues, more specifically in the heart, is likely a result of systemic signaling, e.g. from the liver.

The more quantitative ELISA assay again showed a minor increase in IL-18 levels in the spleen following inhaled administration (Fig. 7C-D). However, overall, results seem to indicate that the inflammatory response to the polymer alone or in TB formulation is minimal. Engulfment of nanoparticles by immune cells in the lung, such as macrophages and dendritic cells, could migrate to the spleen, stimulating inflammasome activation [56, 57]. Finally, TNF-α levels did not show any statistically significant change in any tissue examined (Fig. 7E-F).

## Conclusion

This study demonstrates the potential use of polyelectrolyte surfactant-based nanoparticles as a promising platform for pulmonary delivery of cationic antimicrobials. Our findings show that nanoparticles retain their size and antimicrobial activity following nebulization, while exhibiting high tolerability in both *in vitro* and *in vivo* models. Notably, polyelectrolyte surfactants induce a decrease in mucus viscosity and enhance epithelial cell proliferation, features that may contribute to improved lung penetration and treatment efficacy. Inhaled delivery demonstrated high lung retention with low systemic distribution. Although minor elevations in certain inflammatory markers were observed, overall toxicity remained low. An important consideration in advancing this technology will be achieving dosages sufficient to achieve therapeutic advantage *in vivo*. Nonetheless, these results motivate the further development of polyelectrolyte-based nanocarriers for pulmonary delivery of cationic drug cargoes, particularly in the treatment of chronic lung disease.

## Supplementary Information

Below is the link to the electronic supplementary material.

ESM1 (PDF 327 KB)

## Data Availability

The datasets generated in this study are available from the authors on reasonable request.

## Abbreviations

CF: Cystic fibrosis
CFU: Colony forming unit
ELISA: Enzyme-linked immunosorbent assay
HPLC: High performance liquid chromatography
J: Jeffamine® M-2070
MIC: Minimum inhibitory concentration
MTS: (3-(4,5-Dimethylthiazol-2-yl)-5-(3-carboxymethoxyphenyl)-2-(4-sulfophenyl)-2H-tetrazolium)
NAC: N-acetylcysteine
NP: Nanoparticle
PMAA: Poly(methacrylic acid)
PB: Polymyxin B
PS: Polyelectrolyte surfactant
TSB: Trypticase soy broth
