# Supplementary material for: Aerosol Delivery of Polyelectrolyte Surfactant—Antimicrobial Nanoparticles to the Lungs
Source: Pharm Res. 2026 Jan 9;43(2):421–33. doi: 10.1007/s11095-025-03985-2 (PMC12871378; doi:10.1007/s11095-025-03985-2)
Supplement: Supplementary file 1 — (PDF 327 KB) [file 11095_2025_3985_MOESM1_ESM.pdf]

Aerosol Delivery of Polyelectrolyte Surfactant - Antimicrobial Nanoparticles to the Lungs

Yadiel Varela Soler[1], Amanda S. Padilla-López[2,3], Sughosha Rao[2], Leonardo Calderon[4], Gediminas Mainelis[4], Olga Garbuzenko[5], Tamara Minko[5], David I. Devore[2,6], and Charles M. Roth[1,2,*]

[1]Department of Chemical and Biochemical Engineering, Rutgers University, Piscataway, NJ 08854, USA

[2]Department of Biomedical Engineering, Rutgers University, Piscataway, NJ 08854, USA

[3]Biotechology, University of Puerto Rico, Mayaguez, PR 00680, USA

[4]Department of Environmental Sciences, Rutgers University, New Brunswick, NJ 08901, USA

[5]Department of Pharmaceutics, Rutgers University, Piscataway, NJ 08854, USA

[6]Graplon Technologies, LLC, Langhorne, PA 19047, USA

[*]Correspondence: Department of Biomedical Engineering, 599 Taylor Road, Piscataway, NJ 08854, USA; cmroth@rutgers.edu

Supporting Information

Shear-Viscosity of Artificial Mucus

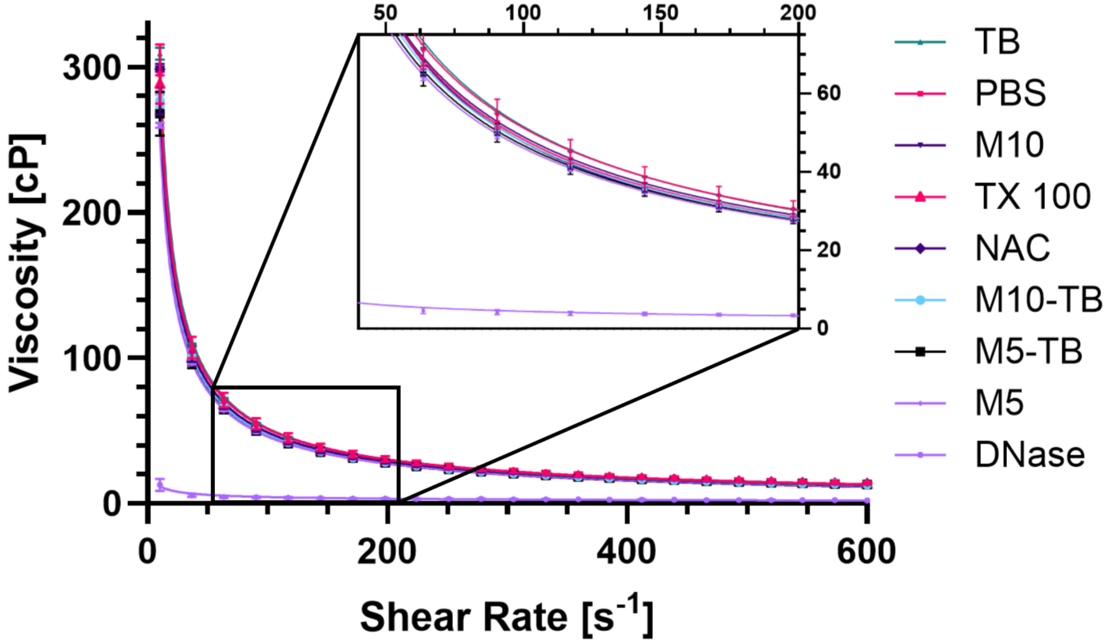

Figure S1. Shear thinning behavior of artificial mucus model when combined with different treatment conditions. Briefly, artificial mucus (1 mL) was combined with different treatment formulations (0.1 mL) and exposed to a shear ramp (10-600 s^{-1}) at 37°C. Treatment solutions included: phosphate buffered saline (PBS), 800 µg/mL tobramycin (TB), 8 mg/mL PMAA-g-10%J (M10), 8 mg/mL PMAA-g-5%J (M5), 10 %v/v Triton X-100 (TX 100), 10 %w/v N-acetylcysteine

(NAC), and 200 U/mL DNase I. M5-TB and M10-TB stand for TB loaded nanoparticles with each respective polymer prepared with a charge ratio of 0.5; their concentration was adjusted so that polymer concentration in the formulation was 8 mg/mL. Curves represent fits of the data to a power law using non-linear regression.

Mass Recovery Following Nebulization

Table S1. Mass-based recovery percentage after nebulization.

	Drug	PMAA-g-10%J
M10+TB	51(±8)%	34(±2)%
M10+PB	70(±9)%	17(±3)%

Relative Distribution of Formulations

Table S2. Relative detected fluorescence for each tissue following administration.

		Liver	Lung	Heart	Kidney	Spleen	Brain
**Inh**	**M10**	2.9 ± 11 %	68 ± 52 %	8 ± 10 %	6.0 ± 7.4 %	8.1 ± 16 %	6.8 ± 6.7 %
	**M10-TB**	0.3 ± 4.6 %	96 ± 69 %	1.6 ± 2.8 %	1.1 ± 3.6 %	0%*	0.9 ± 2.2 %
**IP**	**M10**	65 ± 38 %	1.7 ± 1.7 %	0.9 ± 0.5 %	11 ± 6 %	20 ± 14 %	0.6 ± 0.5 %
	**M10-TB**	53 ± 34 %	4.0 ± 2.8 %	1.5 ± 1.3 %	9.5 ± 6.0 %	31 ± 31 %	1.2 ± 0.5%

** Fluorescence was equal or below to that of the blank.*
Inh and IP stand for Inhalation and intraperitoneal injection.
M10 is Cy5.5 tagged PMAA-g-10%J alone, while M10-TB is nanoparticles loaded with tobramycin.
